# An Exhaustive, Non-Euclidean, Non-Parametric Data Mining Tool for Unraveling the Complexity of Biological Systems – Novel Insights into Malaria

**DOI:** 10.1371/journal.pone.0024085

**Published:** 2011-09-09

**Authors:** Cheikh Loucoubar, Richard Paul, Avner Bar-Hen, Augustin Huret, Adama Tall, Cheikh Sokhna, Jean-François Trape, Alioune Badara Ly, Joseph Faye, Abdoulaye Badiane, Gaoussou Diakhaby, Fatoumata Diène Sarr, Aliou Diop, Anavaj Sakuntabhai, Jean-François Bureau

**Affiliations:** 1 Institut Pasteur, Unité de Pathogénie Virale, Paris, France; 2 Laboratoire de Mathématiques Appliquées Paris 5 (UMR 8145), Université Paris Descartes, Paris, France; 3 Unité d'Épidémiologie des Maladies Infectieuses (UR 172), Institut Pasteur de Dakar, Dakar, Sénégal; 4 Ecole des Hautes Etudes en Santé Publique, Rennes, France; 5 Institute of Health and Science, Paris, France; 6 Unité de Paludologie Afro-Tropicale (UMR 198), Institut de Recherche pour le Développement, Dakar, Sénégal; 7 Laboratoire d'Études et de Recherche en Statistique et Développement, UGB, Saint-Louis, Sénégal; 8 Center of Excellence for Vectors and Vector-Borne Diseases, Faculty of Science, Mahidol University, Bangkok, Thailand; State University of Campinas, Brazil

## Abstract

Complex, high-dimensional data sets pose significant analytical challenges in the post-genomic era. Such data sets are not exclusive to genetic analyses and are also pertinent to epidemiology. There has been considerable effort to develop hypothesis-free data mining and machine learning methodologies. However, current methodologies lack exhaustivity and general applicability. Here we use a novel non-parametric, non-euclidean data mining tool, HyperCube®, to explore exhaustively a complex epidemiological malaria data set by searching for over density of events in m-dimensional space. Hotspots of over density correspond to strings of variables, rules, that determine, in this case, the occurrence of *Plasmodium falciparum* clinical malaria episodes. The data set contained 46,837 outcome events from 1,653 individuals and 34 explanatory variables. The best predictive rule contained 1,689 events from 148 individuals and was defined as: individuals present during 1992–2003, aged 1–5 years old, having hemoglobin AA, and having had previous *Plasmodium malariae* malaria parasite infection ≤10 times. These individuals had 3.71 times more *P. falciparum* clinical malaria episodes than the general population. We validated the rule in two different cohorts. We compared and contrasted the HyperCube® rule with the rules using variables identified by both traditional statistical methods and non-parametric regression tree methods. In addition, we tried all possible sub-stratified quantitative variables. No other model with equal or greater representativity gave a higher Relative Risk. Although three of the four variables in the rule were intuitive, the effect of number of *P. malariae* episodes was not. HyperCube® efficiently sub-stratified quantitative variables to optimize the rule and was able to identify interactions among the variables, tasks not easy to perform using standard data mining methods. Search of local over density in m-dimensional space, explained by easily interpretable rules, is thus seemingly ideal for generating hypotheses for large datasets to unravel the complexity inherent in biological systems.

## Introduction

Identifying the key variables of a biological system that determine the outcome of interest is difficult. Not only are there potentially many factors involved, but they also do not work independently. Testing for all possible interactions is almost impossible both with respect to statistical validation and biological interpretation. There is a need for data mining tools to explore large and complex biological data sets to identify combinations of factors that optimally explain the outcome of interest. Hypothesis-free data exploration can potentially generate novel hypotheses that emerge from the data and which are beyond our imagination. These novel hypotheses can subsequently be tested using standard statistical methods.

To date, data mining tools have been primarily developed for data retrieval through search engines. In biology, this has been essentially focused on sequence alignment algorithms to manage the ever-increasing amount of genetic data. More recently, data mining technology has been proposed as an alternative to traditional statistics to deal with high dimensional data generated by Genome Wide Association studies, in the knowledge that accounting for gene-gene and gene-environment is crucial to understand human genetic susceptibility to disease [1], [2], [3], [4]. In addition to such methods in the field of genetic data analyses, several new heuristic tools have been developed, notably non-parametric modeling techniques such as Classification And Regression Trees (CART) [5] and Random Forests [6]. These methods present several advantages: models have the capacity to provide accurate fits of the response in a wide variety of situations, enabling fitting of non-linear relationships between explanatory variables and the dependant variable, with no assumption that explanatory variables are independent. CART is a rule-based method that generates a binary tree through recursive partitioning. This splits a subset (called a node) of the data set into two subsets (called sub-nodes) according to minimization of a heterogeneity criterion computed on the resulting sub-nodes. Random forests is a procedure that generates a large number of tree predictors and then selects the most popular class. Despite the analytical advances of all of these techniques, none perform exhaustive exploration of the data [4] and to date, there is no algorithm that can search for all possible stratifications and identify the best combination of variables to explain a specified outcome.

Complementary to these non-parametric methods and to traditional statistical methods, a new approach, HyperCube® (Institute of Health & Science, Paris, France) is based on the latest research in artificial intelligence, using least general generalized algorithms and genetic algorithms. The underlying idea is to describe a dataset by a group of « local over densities » of a specific outcome with no *a priori* hypothesis or notion of distance, each « over density » being completely independent from every other. The breakthrough is the ability to deal with points in a space with absolutely no assumptions, including those concerning metric and distance or nature of neighborhood. Indeed, working with a distance or a defined topology is already an assumption and either is not true or introduces bias into the model.

This method has been applied to various topics, mainly in the financial and business sectors, but remains unvalidated in the field of biology [7]. Through exhaustive exploration of m-dimensional space, HyperCube® will classify subsets of the study population into high and low risk groups and pinpoint not only the key explanatory variables and their interactions, but also the key range of values within each explanatory variable. Whilst this approach has evident value for risk factor analysis critical for clinical decision making, it also offers a tool with which to explore complexity, potentially revealing unimaginable combinations of explanatory variables underpinning the observed outcome.

We report here a rigorous assessment of the performance of this novel HyperCube® method. The aim of the study is to test whether the rules identified by HyperCube® give the best predictive value. We use HyperCube® to explore a large longitudinal epidemiological data set of malaria. We compare the predictive value of the rules identified by HyperCube® with models generated using classical statistical methods, binomial regression and CART. We demonstrate that HyperCube® can identify the best combination of factors predicting the outcome of malaria infection in our dataset.

## Results

### Populations, outcome and explanatory variables

We studied a large dataset from a long-term epidemiological study of two family-based cohorts in Senegal, followed for 19 years (1990–2008) in Dielmo and for 16 years (1993–2008) in Ndiop [8], [9]. Time period of observation was classified as a trimester. The dependant variable was defined as a binary trait: individuals with at least one clinical *Plasmodium falciparum* malaria attack (PFA) during that trimester or without PFA. In total, there were 46,837 outcome events of person-trimesters from 1,653 individuals. Almost 20% of the events were PFA in both villages. Thirty-four explanatory variables for association with the occurrence of PFA were considered. Twenty one variables were qualitative (eight nominal and 13 ordered) and 13 were quantitative (Table 1 and 2).

**Table 1:** List of explanatory categorical variables.

Categorical (nominal) Variables	No of levels
House	67 (36 in Dielmo and 31 in Ndiop)
Independent Family	36 (12 in Dielmo and 24 in Ndiop)
Sex	2
Hemoglobin Type	7 (5 in Dielmo and 7 in Ndiop)
ABO blood group	4
G6PD Haplotype (on 4 SNPs: G6PD-376*, G6PD-202*, G6PD-968* and G6PD-542*)	11
PMI	2
POI	2

Categorical (ordered) Variables	No of levels
Drug treatment period	4
Year	19 (19 in Dielmo and 16 in Ndiop)
Trimester	4
ABO-261*: rs8176719	3
ABO-297*: rs8176720	3
ABO-467*: rs1053878	3
ABO-526*: rs7853989	3
ABO-771*: rs8176745	3
Alpha globin-3.7deletion	3
G6PD-202*: rs1050828	3
G6PD-376*: rs1050829	3
G6PD-542*: rs5030872	3
G6PD-968*: rs76723693	3

G6PD: Glucose-6-phosphate dehydrogenase, PMI: *Plasmodium malariae* infection, POI: *Plasmodium ovale* infection.

*: Position on the gene.

**Table 2:** List of explanatory continuous variables.

Continuous Variables	Mean	Median	Min	Max
Age	21.35 (23.14 in Dielmo and 19.46 in Ndiop)	15.90 (17.06 in Dielmo and 14.97 in Ndiop)	0	97.88 (97.88 in Dielmo and 83.25 in Ndiop)
Mean genetic relatedness (Pedigree-based)	0.012 (0.012 in Dielmo and 0.012 in Ndiop)	0.011 (0.012 in Dielmo and 0.008 in Ndiop)	0.001	0.041 (0.028 in Dielmo and 0.041 in Ndiop)
Mean genetic relatedness IBD*-based)	0.008 (0.008 in Dielmo and 0.007 in Ndiop)	0.007 (0.008 in Dielmo and 0.007 in Ndiop)	0.002	0.029 (0.025 in Dielmo and 0.029 in Ndiop)
No. of previous PMI	2.53 (4.10 in Dielmo and 0.82 in Ndiop)	1 (1 in Dielmo and 0 in Ndiop)	0	44 (44 in Dielmo and 9 in Ndiop)
Time since first PMI (year)	6.07 (6.67 in Dielmo and 5.03 in Ndiop)	5.25 (5.95 in Dielmo and4.32 in Ndiop)	0	18.51 (18.51 in Dielmo and 15.25 in Ndiop)
No. of previous POI	1.09 (1.33 in Dielmo and 0.83 in Ndiop)	0	0	11 (11 in Dielmo and 10 in Ndiop)
Time since first POI (year)	5.52 (6.20 in Dielmo and 4.72 in Ndiop)	4.88 (5.55 in Dielmo and 4.25 in Ndiop)	0	18.51 (18.51 in Dielmo and 15 in Ndiop)
Exposure (number of days present in the village) per trimester	80.76 (81.65 in Dielmo and 79.87 in Ndiop)	91 (91 in Dielmo and 90 in Ndiop)	1	92
Distance to animal enclosure (meters)	322 in Dielmo and 147 in Ndiop	271 in Dielmo and 139 in Ndiop	1 in Dielmo and 2 in Ndiop	765 in Dielmo and 393 in Ndiop
Distance to toilets (meters)	326 in Dielmo and 149 in Ndiop	280 in Dielmo and 143 in Ndiop	1 in Dielmo and 2 in Ndiop	774 in Dielmo and 401 in Ndiop
Distance to house's tree (meters)	344 in Dielmo and 152 in Ndiop	311 in Dielmo and 149 in Ndiop	1 in Dielmo and 1 in Ndiop	759 in Dielmo and 386 in Ndiop
Distance to wells (meters)	365 in Dielmo and 195 in Ndiop	453 in Dielmo and 174 in Ndiop	17 in Dielmo and 17 in Ndiop	719 in Dielmo and 483 in Ndiop
Distance to all (animals, toilets, house's tree, wells) together (meters)	329 in Dielmo and 150 in Ndiop	288 in Dielmo and 143 in Ndiop	1 in Dielmo and 1 in Ndiop	774 in Dielmo and 483 in Ndiop

*IBD: Identity-By-Descent.

### HyperCube® analysis

We first analyzed the data using HyperCube®. We divided our dataset into 3 phases: Learning, Validation and Replication. We analyzed the two cohorts separately. A random variable was created dividing the data of each cohort into two groups of equal size (in and out samples). The learning phase was carried out using the “in sample” from the first studied cohort. In the validation phase, rules defined in the learning phase were validated in the “out sample” of the same cohort. The learning set contained 11,893 events and the validation set had 11,939 in Dielmo, while in Ndiop there were 11,530 events in the learning set and 11,475 in the validation set. The effect of each validated rule from the first cohort was studied in the second cohort in the replication phase.

We defined three parameters for running the learning process, “Lift”, “Size” and “Complexity”. “Lift” is the ratio of the prevalence of positive PFA events within a rule over the prevalence of positive PFA events in the entire population; this is equivalent to relative risk (RR). “Size” is the minimum number of events described by the rule. “Complexity” describes the maximum number of variables in a rule. Choice of “Lift” and “Size” parameters are optimized using the “Signal Intensity Graph” (see Material and Method). The “Complexity” parameter is here fixed to six factors, of which two are forced, the “in sample” and the cohort. Table 3 summarizes the parameters used and results obtained from the HyperCube® analyses.

**Table 3:** Parameters used and rules obtained from the HyperCube® analyses.

Cohort	Total number of events	Learning Set	Validation Set	*Purity*	*Lift*	*Size*	Time of run	*Coverage*	Number of Total *rules*	Number of minimized *rules*	Number of validated *rules*	Number of replicated *rules*
Dielmo	23,832	11,893	11,939	0.73	4.00	400	27 h	67%	4,853	52	51	51
Ndiop	23,005	11,530	11,475	0.74	3.49	400	23 h	72%	6,860	36	36	36

Purity: prevalence of events {PFA = 1} in the rule; Lift: Relative Risk of belonging to the rule compared to the total population; Size: number of events in the rule; Coverage: percentage of events {PFA = 1} in all rules found by HyperCube® compared to the total number of events {PFA = 1} in the whole dataset.

**Figure 1:**
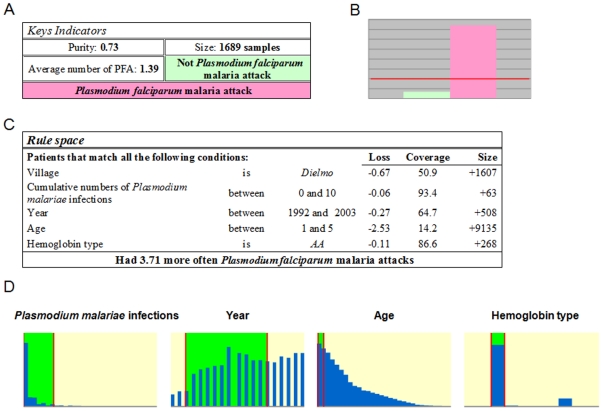
Typical result from HyperCube®. A) Table “*Key Indicators*” shows Lift: 1.39; Size: 1,689; Purity: 0.73. B) Graph showing comparative proportion of events within the rule and events in the entire population, pink: affected (PFA positive), green unaffected (PFA negative). Both pink and green bars would reach the horizontal red line if there was same proportion of positive PFA in the rule and in the entire population. C) Table “*Rule space*” shows marginal contribution of each variable to the lift. Loss: gives partial decreases of lift when removing each variable (or risk factor) from the rule; Coverage: percentage of events {PFA = 1} defined by the corresponding variable alone compared to the total number of events {PFA = 1} in the whole dataset; Size: increase of events in a rule when the constraint defined within a variable is cancelled or by dropping the variable. D) Graphs showing distribution (in blue) of each variable, and the range of values (in green) within the rule.

After 27 and 23 hours of analyses, we obtained 4,853 and 6,860 rules in Dielmo and Ndiop, respectively. We calculated the probability for the occurrence of a rule with identical “Lift” and “Size” parameters from randomization of the entire dataset to obtain an empirical *P* value (emp*P*). We selected minimized rules (see materials and methods) with emp*P* less than 10^−80^ in Dielmo and Ndiop, for the validation phase (Table 3). We used this high threshold emp*P* for selection to minimize the risk of over-fitting. We were able to validate 51 of 52 minimized rules (98%) and 36 of 36 (100%) in Dielmo and Ndiop respectively. Of these, all 51 (100%) rules from Dielmo were replicated in Ndiop and all 36 (100%) rules from Ndiop were replicated in Dielmo with emp*P* less than 10^−3^. We selected the best predicted rule for further statistical study (Figure 1). The best predictive rule contained 1,689 events from 148 individuals and was defined as: individuals who lived in Dielmo during 1992 to 2003, were of an age between 1 to 5 years old, having hemoglobin type AA, and having had previous *Plasmodium malariae* infection (PMI) less than or equal to 10 times. These individuals had 3.71 (95%CI: 3.58–3.84) times more PFA than the general population; and this sub-population was the most representative (i.e. containing the maximum number of events) among those with a RR of at least equal to 3.71.

### Confirmation of the HyperCube® rule with traditional statistical methods

We sought to replicate the HyperCube® rule using logistic regression. We redefined continuous variables as binary variables according to the HyperCube® rule: The “Year” variable was defined as after 1991 and before 2004 or else; Age variable as between 1 and 5 years old or else; Hemoglobin type AA or else and cumulative number of previous PMIs as ≤10 times or else. By multivariate analysis, we tested all possible interactions between two variables and dropped interaction terms with *P*>0.05 until all had *P*≤0.05. The variables showed highly significant marginal effect (*P*<0.0001) except age (Table 4). Age was highly significant (*P*<10^−4^) when taking into account other criteria including year (between 1992 and 2003) and previous PMIs (≤10). Analysis incorporating all possible interaction terms (i.e. with more than 2 variables) generated considerable over-dispersion and was difficult to interpret. This result demonstrates that even though age is a major factor influencing development of PFA, without considering other variables, this effect would have been missed.

**Table 4:** Multivariate analysis of risk factors associated with clinical *P. falciparum* malaria attacks in Dielmo using the HyperCube® rule.

Parameters			DF	Estimate	SE	χ2	Pr>χ2	OR	Wald 95%CL	
Intercept			1	−3.43	0.16	483.4	<.0001	-	-	-
Age group (years)	1 to 5		1	0.38	0.28	1.8	0.178	1.46	[0.84	2.53]
Type of hemoglobin	AA		1	0.38	0.07	27.8	<.0001	1.46	[1.27	1.68]
Year	After 1991 and Before 2004		1	1.80	0.15	139.4	<.0001	6.07	[4.50	8.19]
Number of previous *P. malariae* infections	≤10		1	0.80	0.15	29.4	<.0001	2.23	[1.67	2.97]
Age group *P. malariae infections	1 to 5	≤10	1	1.62	0.27	36.5	<.0001	5.06	[2.99	8.56]
Age group* Year	1 to 5	Before 2004	1	0.77	0.10	55.8	<.0001	2.15	[1.76	2.63]
*P. malariae* infections*Year	≤10	Before 2004	1	−1.38	0.16	72.2	<.0001	0.25	[0.18	0.35]

DF: degree of freedom; Estimate: effect of explanatory variable's levels on *logit*(Probability of {PFA = 1}); SE: standard error; χ2: chi-square DF = 1; OR: Odds ratio; CL: confidential level.

In order to replicate precisely the HyperCube® rule and determine the relative risk for comparison with other models/rules, we estimated the overall effect of the four key variables and all their possible interactions by defining a dummy variable X to represent the two sub groups of the population: X = 1 for a sub-population defined by the observations in the rule (i.e. living in Dielmo during 1992 to 2003, age 1 to 5 years old, having hemoglobin type “AA” and having had previous PMIs≤10); X = 0, otherwise (Table 5). Table 5 shows 1,232 PFA+457 not PFA in the rule = 1,689 events via HyperCube®. The Pearson chi-square test confirmed the strongly significant probability to develop PFA (χ2 = 2740.55, DF = 1, *P*<10^−16^), yielding a RR of 3.71 (95%CI: 3.58–3.84) and odds ratio (OR) of 11.02 (95%CI: 9.87–12.29). Using logistic regression, we confirmed the results of HyperCube®.

**Table 5:** Number of positive/negative PFA events (*P. falciparum* malaria attacks) in subgroups of individuals in and out of the HyperCube® rule.

	PFA positive	No PFA
In the *rule*	1232	457
Out of the *rule*	7977	37171
Total population	9209	37628

### Replication of the rule in the 2^nd^ cohort

In order to validate the biological and epidemiological aspect of this HyperCube® rule, it was replicated in Ndiop where a sub-population defined as above for Dielmo presented a higher risk to develop PFA compared to the general population: (χ2 = 665.96, DF = 1, *P*<10^−16^), RR of 2.35 (95%CI: 2.22–2.48) and OR of 3.50 (95%CI: 3.16–3.87). The result was optimal in Dielmo and replicated in Ndiop. The four variables identified above to be risk factors in Dielmo were thus also risk factors in Ndiop. Keeping the same settings as in Dielmo for time period (from 1992 to 2003), previous PMIs (≤10) and hemoglobin (“AA”), risk was maximum when age was re-set to 3 to 7 years old, with a RR of 2.53 (95%CI: 2.41–2.66) and OR of 4.04 (95%CI: 3.67–4.45) with more events (size = 1,761 events from 181 individuals) and more strongly significant (χ2 = 933.93, DF = 1, *P*<10^−16^) than when using the Dielmo age range of 1–5 years old (Size of 1,607 events from 158 individuals). This risk in Ndiop was, however, still lower than in Dielmo.

The two cohorts differ in one very pertinent manner: in Dielmo malaria transmission occurs all year round because of the presence of a small stream that enables mosquitoes to breed. In Ndiop, transmission is highly seasonal and occurs during the rainy season (July–December). Hence, we calculated the risk in Ndiop using only the period of year between July to December, a period when environmental factors are similar in the two villages. We obtained the same relative risk, RR = 3.78 (95%CI: 3.62–3.94), OR of 11.80 (95%CI: 10.11–13.77), with a highly significant Pearson chi-square test (χ2 = 1542.50, DF = 1, *P*<10^−16^). Furthermore, this risk was maximum when using age 3 to 7 years old (RR = 4.11, 95%CI: 3.97–4.27 and OR = 17.31, 95%CI: 14.68–20.41) with more events (Size = 932 events from 179 individuals *vs.* of Size of 863 from 157 when using age 1 to 5) and higher significance (χ2 = 2076.17, DF = 1, *P*<10^−16^).

### Comparison with other models

We examined whether a classical statistical method could identify the same or better rules. We performed logistic regression analysis and CART using the Dielmo data. We first tested the effect of each variable on PFA by univariate analysis. When two or more variables were correlated, the most explicative variable was chosen. Continuous explanatory variables were categorized to enable comparison with HyperCube®, by grouping the range of values having similar values for the dependant variable. Searching for the cut-off values for continuous variables was guided by Classification and Regression Trees (CART) methods [5]. CART identified cut-off values to categorize Age and Exposure variables, but did not find significant cut-off values for previous PMIs or any other continuous variable. Therefore, median was chosen as the cut-off value for each of these other variables. We then selected variables that showed ≤0.10 type I error for multivariate analysis (Table 6 and 7). As HyperCube® dichotomizes any variable, being in or out of the rule; we redefined each variable in a similar way. Categorical, ordinal and interval variables that had more than 2 levels were redefined by regrouping levels for which their partial effects were in the same direction. Trimester variable was redefined as semester (January–June and July–December) since the first two trimesters had decreasing effects and the last two had increasing effects on PFA when we adjusted on the other variables. Year variable was redefined in two levels (period 1: “year≤2003” and period 2: “year≥2004”) according to the effect of each year. Age variable was classified into two levels (having between 0.4 and 8.1 years-old or else) according to CART analysis, ABO blood group in two levels (O or not O). Table 8 shows the result of univariate analysis after redefinition. For multivariate analysis we used the binary explanatory variables from Tables 6–8 and analyzed by logistic regression using several model selection methods: (1) selection based on an exhaustive screening of candidate models in each subset of explanatory variables, selecting the best one in terms of Information Criterion (lowest Akaike Information Criterion (AIC)); (2) forward selection and backward elimination. Model selection was computed using Package “glmulti” of R software [10]. The results obtained are presented in Table 9.

**Table 6:** Univariate logistic regression analysis of each categorical risk factor for clinical *falciparum* malaria (PFA) attacks in Dielmo.

		No of Person-trimesters						
		N = 23832						
		PFA = 0	PFA = 1	Estimate (Std. Error)	Crude OR	Wald 95%CL	*P*-values	Global *P*
		N(%) = 19475	N (%) = 4357					
Age group (years)	[0–0.4]	303 (84.17)	57 (15.83)	Ref.	1			
	[0.4–6.7]	2344 (46.72)	2673 (53.28)	1.80 (0.15)	6.06	[4.54–8.09]	<.0001	
	[6.7–8.12]	692 (67.13)	338 (32.82)	0.95 (0.16)	2.6	[1.9–3.55]	<.0001	<.0001
	[8.12–13.6]	2943 (81.28)	678 (18.72)	0.20 (0.15)	1.22	[0.91–1.65]	0.1782	
	≥13.6	13138 (95.58)	608 4.42)	−1.40 (0.15)	0.25	[0.18–0.33]	<.0001	
	Missing data	55	3	-	-	-	-	
Sex	Male	9663 (80.77)	2301 (19.23)	Ref.	1			
	Female	9812 (82.68)	2056 (17.32)	−0.13 (0.03)	0.88	[0.82–0.94]	-	<.0001
Blood group	O	7597 (79.56)	1952 (20.44)	Ref.	1			
	A	5131 (83.65)	1003 (16.35)	−0.27 (0.04)	0.76	[0.70–0.83]	<.0001	
	AB	920 (90.20)	100 (9.80)	−0.86 (0.11)	0.42	[0.34–0.52]	<.0001	<.0001
	B	4496 (82.40)	960 (17.60)	−0.19 (0.04)	0.83	[0.76–0.91]	<.0001	
	Missing data	1331	342	-	-	-	-	
Type of hemoglobin	AA	16304 (81.28)	3756 (18.72)	Ref.	1			
	AC/AS/SS	2007 (87.53)	286 (12.47)	−0.48 (0.07)	0.62	[0.54–0.70]		<.0001
	Missing data	5196	1438	-	-	-	-	
G6PD	Normal alleles	6448 (84.0)	1228 (16.0)	Ref.	1			
	Mutated allele	7865 (82.30)	1691 (17.70)	−0.12 (0.04)	0.89	[0.82–0.96]		0.0032
	Missing data	5162	1438	-	-	-	-	
*P. malariae* infections	≤1 (median)	9348 (81.99)	2099 (18.34)	Ref.	1			
	>1	8983 (79.91)	2258 (20.09)	0.11 (0.03)	1.12	[1.04–1.20]	-	0.0008
	missing	1144	0	-	-	-		
*P. ovale* infections	≤0 (median)	9946 (81.54)	2251 (18.46)	Ref.	1			
	>0	8385 (79.93)	2106 (20.07)	0.10 (0.03)	1.11	[1.04–1.19]	-	0.002
	missing	1144	0	-	-	-		

Estimate: effect of explanatory variable's levels on *logit*(Probability of {PFA = 1}); SE: standard error; OR: Odds ratio; CL: confidential level; Ref.: reference level.

Age and Exposure were categorized using CART and previous PMIs and previous POIs using median since CART did not find significant cut-off values.

**Table 7 pone-0024085-t007:** Univariate logistic regression analysis of each temporal risk factor for clinical *falciparum* malaria (PFA) attacks in Dielmo.

		No of Person-trimesters						
		N = 23832						
		PFA = 0	PFA = 1	Estimate (Std. Error)	Crude OR	Wald 95%CL	*P*-values	Global *P*
		N(%) = 19475	N (%) = 4357					
Year	1990	587 (82.21)	127 (17.79)	Ref.	1			
	1991	740 (81.59)	167 (18.41)	0.04 (0.13)	1.04	[0.81–1.35]	0.7457	
	1992	717 (77.18)	212 (22.82)	0.31 (0.13)	1.37	[1.07–1.75]	0.0126	
	1993	790 (78.61)	215 (21.39)	0.23 (0.12)	1.26	[0.99–1.61]	0.0653	
	1994	774 (75.44)	252 (24.56)	0.41 (0.12)	1.50	[1.19–1.91]	0.0008	
	1995	796 (77.06)	237 (22.94)	0.32 (0.12)	1.38	[1.08–1.75]	0.0093	
	1996	853 (72.23)	328 (27.77)	0.58 (0.12)	1.78	[1.41–2.24]	<.0001	
	1997	818 (73.3)	298 (26.7)	0.52 (0.12)	1.68	[1.33–2.13]	<.0001	
	1998	1179 (80.2)	291 (19.8)	0.13 (0.12)	1.14	[0.91–1.44]	0.2632	
	1999	1137 (78.09)	319 (21.91)	0.26 (0.12)	1.30	[1.03–1.63]	0.0258	<.0001
	2000	1151 (76.84)	347 (23.16)	0.33 (0.12)	1.39	[1.11–1.75]	0.0041	
	2001	1019 (77.91)	289 (22.09)	0.27 (0.12)	1.31	[1.04–1.65]	0.0222	
	2002	1061 (80.75)	253 (19.25)	0.1 (0.12)	1.10	[0.87–1.40]	0.4188	
	2003	1055 (80.47)	256 (19.53)	0.11 (0.12)	1.12	[0.89–1.42]	0.3396	
	2004	1153 (87.81)	160 (12.19)	−0.44 (0.13)	0.64	[0.50–0.83]	0.0006	
	2005	1312 (91.11)	128 (8.89)	−0.8 (0.13)	0.45	[0.35–0.59]	<.0001	
	2006	1228 (83.2)	248 (16.8)	−0.07 (0.12)	0.93	[0.74–1.18]	0.5663	
	2007	1495 (90.44)	158 (9.56)	−0.72 (0.13)	0.49	[0.38–0.63]	<.0001	
	2008	1610 (95.72)	72 (4.28)	−1.58 (0.16)	0.21	[0.15–0.28]	<.0001	
Season	Jan–Mar	4749 (82.62)	999 (17.38)	Ref.	1			
	April–June	4912 (82.03)	1076 (17.97)	0.04 (0.05)	1.04	[0.95–1.14]	0.4029	
	July–Sept	4841 (80.38)	1182 (19.62)	0.15 (0.05)	1.16	[1.06–1.27]	0.0017	0.0128
	Oct–Dec	4973 (81.89)	1100 (18.11)	0.05 (0.05)	1.05	[0.96–1.16]	0.2973	
Exposure	≤66.5 days	2978 (94.33)	179 (5.67)	Ref.	1			
	>66.5 days	15745 (81.57)	3558 (18.43)	1.32 (0.08)	3.76	[3.22–4.39]	-	<.0001
		752	620	-	-	-		

Estimate: effect of explanatory variable's levels on *logit*(Probability of {PFA = 1}); SE: standard error; OR: Odds ratio; CL: confidential level; Ref.: reference level.

Age and Exposure were categorized using CART and previous PMIs and previous POIs using median since CART did not find significant cut-off values.

**Table 8:** Univariate analysis of each risk factor (redefined in only two levels) for clinical *P. falciparum* malaria attacks (PFA) in Dielmo.

		No of Person-trimesters					
		N = 23832					
		PFA = 0	PFA = 1	Estimate (Std. Error)	Crude OR	Wald 95%CL	*P*-values
		N (%) = 19475	N (%) = 4357				
		(81.72)	(18.28)				
Age group (years)	<0.4 or ≥8.12	16384 (92.42)	1343 (7.58)	Ref.	1		
	[0.4–8.12]	3036 (50.21)	3011 (49.79)	2.49 (0.04)	12.1	[11.22–13.04]	<.0001
	Missing data	55	3	-	-	-	
Blood group	A or B or AB	10547 (83.64)	2063 (16.36)	Ref.	1		
	O	7597 (79.56)	1952 (20.44)	0.27 (0.04)	1.31	[1.23–1.41]	<.0001
	Missing data	1331	342	-	-	-	
Year	≥2004	6798 (89.87)	766 (10.13)	Ref.	1		
	<2004	12677 (77.93)	3591 (22.07)	0.92 (0.04)	2.51	[2.31–2.73]	<.0001
Semester	Jan–Jun	9661 (82.32)	2075 (17.68)	Ref.	1		
	Jul–Dec	9814 (81.13)	2282 (18.87)	0.08 (0.03)	1.08	[1.16–1.16]	0.0179

Estimate: effect of explanatory variable's levels on *logit*(Probability of {PFA = 1}); SE: standard error; OR: Odds ratio; CL: confidential level; Ref.: reference level.

**Table 9:** Multivariate model selection for risk factors associated with clinical *P. falciparum* malaria attacks (PFA) in Dielmo using factors identified from univariate logistic analysis.

		Best model (with lowest AIC) when number of explanatory variables is equal to:											
Variables		1	2	3	4	5	6	7	8	9	10	Forward*	Backward*
Sex	Female										√	NSE	1
Age group (years)	0.4 to 8.1	√	√	√	√	√	√	√	√	√	√	1	NSR
Blood Type	O									√	√	9	NSR
Type of hemoglobin	AA							√	√	√	√	7	NSR
G6PD	Normal					√	√	√	√	√	√	6	NSR
Year	Before 2004		√	√	√	√	√	√	√	√	√	2	NSR
Semester	Jul–Dec								√	√	√	8	NSR
Exposure	>66.5				√	√	√	√	√	√	√	4	NSR
*P.malariae* infections	≤1			√	√	√	√	√	√	√	√	3	NSR
*P.ovale* infections	≤0						√	√	√	√	√	5	NSR
RR		2.53	2.96	3.22	3.22	3.15	2.98	3.08	3.27	3.32	2.95	3.32	3.32
(95% CI)		(2.45–2.61)	(2.86–3.05)	(3.10–3.35)	(3.09–3.37)	(2.93–3.38)	(2.71–3.27)	(2.79–3.40)	(2.89–3.70)	(2.83–3.91)	(2.18–3.99)	(2.83–3.91)	(2.83–3.91)
p-value		<.0001	<.0001	<.0001	<.0001	<.0001	<.0001	<.0001	<.0001	<.0001	<.0001	<.0001	<.0001
Size of subset defined by all risk factors		6044	4277	2000	1520	507	316	261	143	78	31	78	78

√ : For selected variables.

NSE: No (additional) effects met the 0.05 significance level for entry into the model.

NSR: No (additional) effects met the 0.05 significance level for removal from the model.

*: Both Forward and Backward methods selected the best (in terms of AIC) model with 9 explanatory variables.

According to the results of the multivariate regression model selection (Table 9), we defined for each selected model a sub-group X = 1 when all risk factors are present, otherwise X = 0. For each model, we gave RR, p-value, and number of events for the sub-group having all identified risk factors. All sub-groups identified using model selection techniques had lower predictive values for developing PFA than the HyperCube® rule (Table 9). For sub-groups explaining the same or a greater number of events than the one found by HyperCube®, the RR was lower and the 95% confidential intervals of RR did not overlap with those for the HyperCube® rule (Table 9).

We tested whether the HyperCube® rule predicted the highest risk of developing PFA. We used the HyperCube® model as a reference. We modified the reference HyperCube® rule by either removing one of the variables or adding in variables identified by multivariate analysis. Using the same method to define subsets of the population and construct contingency tables, we calculated RR, OR and *P* values for each model. As shown in Table 10, there was no other model that gave higher RR and/or OR than the one identified by HyperCube® with equal or greater size.

**Table 10:** Predictive values of modified HyperCube® rule.

Variable	Size	RR	95%CL		OR	95%CL		χ^2^	DF	Pr>χ^2^
M.ref:		3.71	3.58	3.84	11.02	9.87	12.29	2741	1	<.0001
*P. malariae* infections+Year+Age+Hemoglobin	**1689**									
M.ref−*P. malariae* infections	**1752**	3.65	3.52	3.77	10.35	9.30	11.51	2705	1	<.0001
M.ref−Year	**2197**	3.44	3.33	3.56	8.58	7.82	9.40	2843	1	<.0001
M.ref−Age	**10824**	1.18	1.14	1.23	1.24	1.18	1.30	71	1	<.0001
M.ref−Hemoglobin	**1957**	3.60	3.48	3.73	9.94	9.00	10.99	2898	1	<.0001
M.ref+Sex−*P. malariae* infections	879	3.69	3.53	3.86	10.82	9.31	12.57	1475	1	<.0001
M.ref+Sex−Year	1031	3.59	3.44	3.75	9.82	8.57	11.25	1592	1	<.0001
M.ref+Sex−Age	**5377**	1.16	1.10	1.22	1.20	1.13	1.29	29	1	<.0001
M.ref+Sex−Hemoglobin	990	3.62	3.46	3.78	10.06	8.75	11.56	1562	1	<.0001
M.ref+Blood Type−*P. malariae* infections	784	3.61	3.44	3.79	10.03	8.58	11.72	1249	1	<.0001
M.ref+Blood Type−Year	966	3.46	3.30	3.63	8.69	7.57	9.96	1351	1	<.0001
M.ref+Blood Type−Age	**4312**	1.29	1.22	1.36	1.38	1.29	1.49	78	1	<.0001
M.ref+Blood Type−Hemoglobin	852	3.66	3.50	3.83	10.48	9.01	12.19	1399	1	<.0001
M.ref+G6PD−*P. malariae* infections	651	3.76	3.58	3.95	11.56	9.69	13.79	1162	1	<.0001
M.ref+G6PD−Year	717	3.72	3.55	3.91	11.17	9.46	13.20	1244	1	<.0001
M.ref+G6PD−Age	**4840**	1.17	1.11	1.23	1.22	1.13	1.31	30	1	<.0001
M.ref+G6PD−Hemoglobin	661	3.84	3.66	4.02	12.59	10.53	15.05	1249	1	<.0001
M.ref+Semester−*P. malariae* infections	884	3.77	3.62	3.94	11.76	10.09	13.69	1574	1	<.0001
M.ref+Semester−Year	1117	3.56	3.41	3.72	9.54	8.38	10.86	1677	1	<.0001
M.ref+Semester−Age	**5458**	1.23	1.17	1.30	1.31	1.23	1.40	64	1	<.0001
M.ref+Semester−Hemoglobin	988	3.76	3.61	3.92	11.62	10.06	13.42	1734	1	<.0001
M.ref+Exposure−*P. malariae* infections	1403	3.66	3.25	3.80	10.46	9.29	11.78	2228	1	<.0001
M.ref+Exposure−Year	**1804**	3.44	3.31	3.57	8.51	7.69	9.42	2367	1	<.0001
M.ref+Exposure−Age	**8729**	1.15	1.11	1.20	1.20	1.14	1.27	42	1	<.0001
M.ref+Exposure−Hemoglobin	1535	3.62	3.49	3.76	10.14	9.05	11.35	2361	1	<.0001
M.ref+*P. ovale* infections−*P. malariae* infections	729	3.88	3.71	4.06	13.13	11.06	15.60	1410	1	<.0001
M.ref+*P. ovale* infections−Year	759	3.87	3.71	4.05	13.05	11.02	15.44	1459	1	<.0001
M.ref+*P. ovale* infections−Age	**4256**	1.52	1.44	1.59	1.73	1.62	1.86	246	1	<.0001
M.ref+*P. ovale* infections−Hemoglobin	768	3.85	3.69	4.03	12.79	10.82	15.10	1456	1	<.0001

M.ref: reference model; Size: number of events; RR: risk ratio; OR: Odds ratio; χ2: chi-square DF = 1; CL: confidential level.

**Figure 2:**
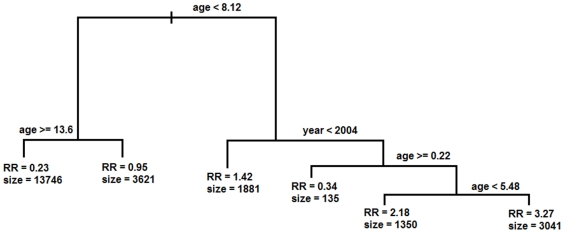
Decision tree generated by Classification and Regression Tree (CART) analysis of risk factors determining the occurrence of *P. falciparum* malaria attacks (PFA) per trimester. Figure shows the cut-off values identified by CART that divide the dataset into two. At each leaf are given the Relative Risk (RR) and the number of events associated with that leaf.

In contrast to the regression analyses, CART found that age (between 0.22 and 5.48) and year (from 1990 to 2003) defined the high risk group for having PFA (RR = 3.26 [95CI: 3.16–3.38], OR = 7.34 [95CI: 6.80–7.93] and size = 3,041 with χ2 = 3268.85, DF = 1 [P<10^−16^]) (Figure 2). No other variable or combination of variables yielded a higher Relative Risk.

### Optimality of HyperCube® choice

**Figure 3:**
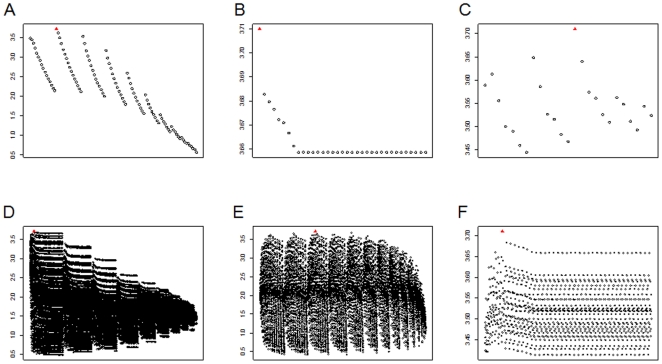
Effect on relative risk (RR) of modifying the ranges of continuous variables. Graphs show RR for all other possible definitions of risk group on the explanatory variables, with equal or greater size than the HyperCube® rule. Y-axis indicates the RR. A) Only ranges of Age are modified: 102 choices among 4,851 possible choices had size equal or greater than 1,689 (size of the HyperCube® rule) and are plotted; B) Only ranges of previous PMIs are modified: 35 choices among 1,035 possible; C) Only ranges of Year are modified: 25 choices among 190 possible; D) Ranges of both Age and previous PMIs are modified simultaneously: 25,040 choices among 5,020,785 possible; E) Ranges of both Age and Year are modified simultaneously: 8,912 choices among 921,690 possible; F) Ranges of both previous PMIs and Year are modified simultaneously: 1,110 choices among 196,650 possible. Filled red triangle represents the RR of HyperCube®'s rule (HyperCube®'s risk group), empty black circles represent the RR of other choices of risk groups.

We then tested whether the cut-off values delimiting the range of values in the HyperCube® rule (defined as the reference rule) for each variable were the optimal ones. Hemoglobin type was fixed as AA or not. We modified the range of continuous variables of the reference rule. As the cut-off values for continuous variables were considered at integer values, there were a finite number of subsets that we could try for modifying a rule. We tested all possible ranges of the continuous variables (Age, previous PMIs and Year) with constraint of minimum “Size” of ≥400 events in the rules. We first fixed 2 variables and changed one variable at a time. The variable to change was first defined as the range of integer values between its minimum and maximum values, and then reduced from the maximum to smaller integer values covering an ever-decreasing total age range until the minimum. This was repeated step by step until each integer value of the variable was set as the minimum for a step. Therefore, the total number of choices for a variable is 1+2+3+…+*maximum* = sum of a finite arithmetic sequence = (first value+last value)*(number of values)*(1/2). Each choice corresponds to a specific modification of the reference rule (i.e. a specific interval of values defining the modified rule). Then, for Age, previous PMIs and Year, there are (1+98)*98*0.5 = 4851, (1+45)*45*0.5 = 1035 and (1+19)*19*0.5 = 190 possible choices respectively. We then fixed 1 variable and changed 2 variables simultaneously. When Year is fixed and the couple (Age, previous PMIs) changed simultaneously, there are 4851*1035 = 5,020,785 possible choices. For previous PMIs fixed and (Age, Year) changed and Age fixed and (previous PMIs, Year) changed there are 4851*190 = 921,690 and 1035*190 = 196,650 possible choices. When we selected choices with at least same size as the reference rule, the resulting RR was always lower than the reference RR. Figure 3 shows the effects of the modified ranges (i.e. the effect of other choices different from the one found by HyperCube®) on RR. If all 3 variables were allowed to vary simultaneously there would be 4,851(Age) *190(Year) *1035(previous PMIs) = 953,949,150 possible choices. The time for running such an analysis on one computer with 2 central processor units (Duo CPU 2.00 GHz 2.00 GHz), Memory (RAM) of 3.00 GB) is estimated at ∼5678 days (∼1.94 choices analyzed per second) using function “*system.time(.)*” of R-software, and thus not possible to analyze.

## Discussion

We describe here a new data mining algorithm that can identify the combinations of variables that give the optimal prediction of the outcome of interest. We demonstrate that the model identified by HyperCube® has better predictive value than any other model tested. HyperCube® was able to identify the best cut-off value and range for continuous variables. It classified the population into high and low risk groups and made the results easier to interpret in terms of biology than the probability estimates generated by most statistical methods.

The principle of this method is to explore all possible combinations of predictor variables and to find, through stochastic parallel computing exploration, the optimal hypercubes (or sub-spaces) defined by a combination of these variables, without making any assumptions. This method allows generation of rules, sets of variables and ranges of variable values that define subpopulations with high risk for the outcome of interest and that best predict the outcome. Inspired from latest research in artificial intelligence, Least General Generalized algorithms and Genetic Algorithms, HyperCube® SaaS software generates local hypercubes and stabilizes each local hypercube to a local optimum, each optimum being new and independent. By doing so, it is possible to describe and understand local configurations without there being necessarily any global effect, i.e. some specific combination of factors that are only found in a sub-set of the population may increase the risk of outcome for that sub-population, but which are not detectable when averaged across the entire population. HyperCube® enables us to describe the range of values and the combination of variables that can trigger the events. Although the statistics aims to reject, or not, a predefined assumption according to given risks, these complex event intelligence techniques allow us to generate assumptions on rules without any prerequisite. A hypercube is expressed in a simple formal way as a rule, directly readable and comprehensible.

As correction for multiple testing is not possible when using HyperCube®, statistical validation and replication in independent cohorts are crucial, even prior to biological validation. We randomly divided the population in one cohort into the learning set and the validation set. We used the other cohort for replication. In addition, we calculated an empirical *P* value from whole randomized data. We demonstrated that using a high threshold of empirical *P* value (10^−80^), 98–100% of the rules could be validated and 100% of validated rules could be replicated in another cohort despite their differences in human ethnicity and malaria endemicity [11].

Biological validation of the rule is most important. Here three of the variables are known *a priori* to increase the risk of developing PFA: young children (i.e. lack of clinical immunity), normal hemoglobin Hb AA, and living during a period of intense malaria transmission. However, HyperCube® allowed us to identify the range of continuous variables, such as age and year, which enable us to define high and low risk groups. In addition, the effect of these three variables alone did not reach our stringent acceptance threshold. Identifying an additional variable using classical techniques would be a big challenge due to the number of possible choices. HyperCube® added a fourth one “number of previous PMIs at ranges less than or equal to 10” to define a rule containing 1,232 events with PFA and 457 events without PFA (prevalence = 72.9%) compared to 19.7% prevalence of the whole population (RR 3.71 (95%CI: 3.58–3.84). This RR is the highest of all models containing this number of events. This rule explained 28.28% of total events with PFA in the dataset.

The effect size of each variable was estimated by removing each variable and calculating the loss in “Lift” (Figure 1c). The strongest effect is age (68%), then village (18%), followed by year (7.3%). Hemoglobin type explained 3% of the “Lift” while previous PMIs had only 1.6% effect. There was 1.8% of the “Lift” that could not be explained by each of these variables individually (Table 11) and thus reflects interaction among the variables. In Dielmo, malaria transmission is holoendemic with an average of more than 200 infectious bites per person per year, 10 times more than Ndiop [12]. Therefore, individuals living in Dielmo have more chance to develop PFA. Age is a well known factor of PFA due to rapid development of clinical immunity in high malaria transmission regions. Using variance component analysis, age explained 29.8% of total variation in number of PFA in Dielmo [11]. The year effect is almost certainly yearly variation in transmission intensity. Indeed in 2003, the HyperCube® rule threshold for year, a new drug for PFA treatment was introduced and malaria transmission decreased in following years. Hemoglobin type is one of the best known genetic factors protecting against malaria. In our and other studies, sickle cell mutation explained 2–5% of risk in development of severe and clinical *falciparum* malaria [13], similar to that estimated by HyperCube® (Table 11). The new variable that HyperCube® identified is previous *P. malariae* infection - PMI. Although CART did not identify any significant threshold for previous PMI, using the median as the cut-off value gave a significant effect for previous PMI is the univariate logistic regression, whereby above median previous PMI increased risk of PFA (P = 0.0008, Table 6). Interestingly in the HyperCube® rule the reverse was found and this is because of the interaction of previous PMI with age: being young and having previous PMI decreased risk.

**Table 11:** Effect size of each variable in the rule.

	DIELMO		NDIOP			
			All year		July	December
	Loss	% Loss	Loss	% Loss	Loss	% Loss
**Initial Lift**	3.71	100%	2.35	100%	3.78	100%
**Age**	−2.53	−68.2%	−0.82	−34.9%	−1.26	−33.3%
**Village**	−0.67	−18.1%	−0.7	−29.8%	0.05	1.3%
**Year**	−0.27	−7.3%	−0.07	−3.0%	−0.06	−1.6%
**Hb**	−0.11	−3.0%	−7.0%	−3.0%	−0.09	−2.4%
**Previous PMIs**	−0.06	−1.6%	−0.13	−5.5%	−0.12	−3.2%
**Semester**	-	-	-	-	−1.43	−37.8%
**Total Loss**	−3.64	−98%	−1.79	−76%	−2.91	−77%
**Residual Lift**	0.07	1.9%	0.56	23.8%	0.87	23.0%

Loss: partial decreases of lift when removing each variable from the rule.

Cross-species immunity among different *Plasmodium* species has long been suspected and there is evidence of among-species negative interactions during concomitant infection [14], [15]. An influence of *P. malariae* carriage on subsequent *P. falciparum* infection has been observed before. In Gabon, children infected with *P. malariae* presented more often with a *P. falciparum* infection and at higher parasite densities [16]. During the follow-up, subjects who were infected by *P. malariae* were reinfected by *P. falciparum* more rapidly. Such a relationship was also observed in the Garki project [15], [17], [18]. Although small scale variation in mosquito biting rate could generate similar levels of exposure to each parasite spp., the species infection association was found to be related to differences in acquired immunity and not to differences in exposure, suggesting that the levels of immunity to *P. falciparum* and to *P. malariae* were inter-related [18]. More recently, a family-based study found a strong relationship between *P. falciparum* parasite density and frequency of *P. malariae* infections [19]. *P. falciparum* parasite density has previously been shown to be under human genetic control and linked to the chromosomal region 5q31 in four independent studies [11], [20], [21], [22]. These results suggest that individuals genetically susceptible to *P. falciparum* are also genetically pre-disposed to *P. malariae* [19]. Little is known on the impact of infection by one species on the incidence of disease of another. The relationships between parasite density and risk of attributable disease were found to be similar for *P. falciparum*, *P. vivax* and *P. malariae* in Papua New Guinea, compatible with the hypothesis that pan-specific mechanisms may regulate tolerance to different *Plasmodium* spp. [23]. Pertinent to our finding here, Black *et al.* found that children with symptomatic episodes not only presented with fewer mixed species infections, but also had fewer previous *P. malariae* infections than symptom-free children, as demonstrated by serology [24]. The induced infection experiments also provide evidence of the development of some cross-protective immunity [25]. Interestingly, previous infection with *P. malariae* has been previously shown to impact upon a *P. falciparum* infection, but with respect to the production of transmission stages and not clinical presentation [26], [27].

Many other rules used this variable confirming that previous infection by *P. malariae* is associated with protection against development of PFA. It is presently impossible to conclude if this association is a causal one or is due to a correlation to an unknown factor affecting the risk to develop PFA. As both parasites are transmitted by the same mosquito species, increased exposure to one species (*P. malariae*) might be expected to correlate with increased exposure to the other (*P. falciparum*). Hence, spatial heterogeneity in the exposure to infection could simultaneously result in increase risk of infection by both parasite *spp*. Our analysis did not take into account “number of previous *P. falciparum* attacks” (nbpPFA) and so it is possible that the variable previous PMIs replaces this information. However, in another HyperCube® analysis, we found that both previous PMIs and nbpPFA are used in different rules (data not shown), indicating that the previous infection by the two parasite species is not perfectly correlated. Thus, it seems probable that the parasite species effect reflects some impact of *P. malariae* infection on the development of immunity against *P. falciparum*. In our study, there were from 0 to 44 *P. malariae* infections per person prior to a clinical *P. falciparum* episode. Hypercube® identified that having few *P. malariae* infections (less than 10) was a potent risk factor, which excluded about 10% of events from those individuals who were often infected with *P. malariae*. The fact that a threshold of ten infections was identified as eliminating this risk factor is clearly not an exact threshold, but generally reflects the weakly immunising effect of *P. malariae* infection, reminiscent of that induced by *P. falciparum* infection. Furthermore, whereas eighteen out of 51 rules used the number of previous *P. malariae* infections, none used the number of previous *P. ovale* infections, illustrating that infection by the two *Plasmodium* species differently affects susceptibility to *P. falciparum* attacks. However, it should be noted that the absence of an effect of *P. ovale* on clinical *P. falciparum* attacks does not mean that *P. ovale* definitively has no effect. It may be the case that additional variables may be required to be taken into account. Indeed, in the multivariate model selection analysis (Table 9), previous P. ovale infection is significantly as a risk factor when a minimum of 6 explanatory variables are used. In our HyperCube® analyses, we limited the number of variables in a single rule to four. This differential species effect is currently under investigation.

We compared the rule with the model identified by classical logistic regression method. Although we aimed to include all possible interaction terms among variables studied in multivariate analysis, over-dispersion of the data made this unstable. In addition, the running time would have been unacceptably long, taking ∼5678 days for one a common computer to analyze about 10^9^ models (3 variables with around 10^3^ cases for each). With HyperCube®, it took 23 to 27 hours to analyze 35 variables. In addition, the results of testing interaction among more than 2 variables by classical methods are difficult to interpret. We demonstrated that by omitting or adding other variables identified by other statistical methods or varying the cut-off value of continuous variables, the rule still performed best. Although some rules had higher RR, they have lower “Size” or more complexity and less significant *P* value. Among rules with “Size” equal to or greater than 1,689, the same as the reference rule, the reference rule gave the highest RR.

Interestingly, the rule identified by classical method covered 0.67% of total positive events whereas one HyperCube® rule explained 13.4%. When considering the minimized rules, we could identify risk factors that could explain 67% of total positive events, a percentage of coverage that would never be achieved by classical methods. While the classical method looked at events in 2 dimensions, HyperCube® identified rules in multi-dimensional space. Although all factors identified by the classical method are risk factors for development of PFA, different groups of people developed PFA for different reasons. The rule identified by the classical method involved only individuals who had all the risk factors. We could only separate groups of individuals with different risk factors when looking at the events in multi-dimensional space.

Analysis by CART identified a combination of variables, Age and Year, that increased risk of PFA. Both of these variables and the range of these variables were very similar to those identified by HyperCube®. That CART failed to detect Hemoglobin or previous PMIs likely reflects the differences in methodologies of the two techniques. CART uses a sequential approach first splitting the data set according to the most significant variable and identifying the threshold value of that variable that maximizes the discrimination in the two subsets of data (i.e. least PFA *vs.* most PFA). Then, CART will further sub-divide each subset by the next most significant variable that leads to maximum discrimination. This approach thus leads to canalization of the data along different pathways, resulting in a decreased sample size for comparison. In addition, optimization by maximum discrimination at each level may paradoxically lead to an erroneous sub-optimal end-point many levels down. HyperCube®, by contrast, analyses all variables simultaneously with no sequential selection that leads to such loss of power or canalization along a potentially eventual sub-optimal pathway.

One limitation arises when studying qualitative variables with more than two levels. It is not possible for HyperCube® to combine levels having a similar effect in the same rule. One alternative would be to use analysis of variance, as we previously did in our classical analysis for qualitative variables with more than 2 levels, to detect modalities having a similar effect on the dependant variable and group them *a priori*.

Another more practical problem comes from the efficiency of the learning process. This process is more efficient in explaining the minor outcome, which is sometimes not the standard way of thinking. For instance, we could identify only factors increasing the risk of PFA, but not those conferring protection against malaria, which is the classical choice in malaria field. The positive events for PFA made up ∼15% of the total number of events. To identify factors conferring protection (negative PFA), of which the prevalence was 85%, would have presented a vastly increased analytical challenge and yielded many, many more rules.

The choice of minimum group size for the outcome variable can, however, generate problems for biological interpretation. For example, here we observe that hemoglobin AA (normal hemoglobin) increases risk for development of PFA compared to the mutated sickle form, AS, which is known to confer protection. Importantly, we cannot conclude from our analysis that AS confers protection. In general, care must be taken in interpreting the direction of the effect and further specific analyses should be performed prior to establishing formal conclusions.

Repeated measures and potential pseudo-replication of events from the same individual are difficult to take into account. Whilst this can be accounted for *a posteriori* in confirmatory classical analyses, this cannot be currently taken into account in HyperCube®. For the rules obtained, the full information on the number of events and the number of people contributing to those events can be provided, as done here. In addition, with regard to use of human genetic factors as explanatory variables, bias due to population stratification is difficult to take into account in HyperCube®. Such a bias needs to be secondarily tested on validated rules using classical methods.

A final limitation is that HyperCube® requires huge computational power and needs to use massive parallel processing. Today, HyperCube® is accessible as a web based software that requires no specific learning skills, though it requires significant computing power provided through SaaS architecture. Currently HyperCube® is used on various complex problems [7]; we now report an analysis of epidemiological data using this algorithm. HyperCube® classified events or individuals into high and low risk groups defined by combinations of variables. It efficiently sub-stratified quantitative variables to optimize the effect. In addition, it was able to identify interactions among the variables. These tasks are not easy to perform using standard data mining methods. HyperCube® is very useful in handling large datasets with complexity of the dependant variable, such as found in large epidemiological studies and genetic studies. We have proved that the rules identified by HyperCube® are the optimal in the dataset and that no other methods can find them in a reasonable time. Search of local over density in m-dimensional space, explained by easily interpretable rules, is thus seemingly ideal for generating hypotheses for large datasets to unravel the complexity inherent in biological systems. Hypotheses generated by this data mining program should be validated using classical statistical methods and/or by biological experimentation. Further statistical analyses, to provide adequate description and inference on the sub-population identified in a rule, have to be performed by using specific models (e.g. Generalized Estimating Equations [28] or Generalized Linear Mixed Models [29] to take into account repeated measures and/or genetic covariance between individuals, or distribution of the dependent variable).

## Materials and Methods

### Ethics statement

The project protocol and objectives were carefully explained to the assembled village population and informed consent was individually obtained from all subjects either by signature or by thumbprint on a voluntary consent form written in both French and in Wolof, the local language. Consent was obtained in the presence of the school director, an independent witness. For very young children, parents or designated tutors signed on their behalf. The protocol was approved by the Ethical Committee of the Pasteur Institute of Dakar and the Ministry of Health of Senegal. An agreement between Institut Pasteur de Dakar, Institut de Recherche pour le Développement and the Ministère de la Santé et de la Prévention of Senegal defines all research activities in the study cohorts. Each year, the project was re-examined by the Conseil de Perfectionnement de l'Institut Pasteur de Dakar and the assembled village population; informed consent was individually renewed from all subjects.

### Populations

The populations studied come from two family-based village cohorts, Dielmo and Ndiop, in Senegal. These populations have been recruited for a long-term immunological and epidemiological study [8]. Malaria transmission intensity differs between the 2 villages because of the presence of the river in one of them that offers a mosquito breeding site all-year round.

Research stations have been installed in the villages with full-time nurses and paramedical personnel. Almost all fever episodes were reported to the clinics with blood smears checked for malaria parasites. The outcome of interest is a *Plasmodium falciparum* malaria attack (PFA). PFA was defined as a presentation with measured fever (axillary temperature >37.5°C) or fever-related symptoms (headache, vomiting, subjective sensation of fever) associated with i) a *P. falciparum* parasite/leukocyte ratio higher than an age-dependent pyrogenic threshold previously identified in the patients from Dielmo [30], ii) a *P. falciparum* parasite/leukocyte ratio higher than 0.3 parasite/leukocyte in Ndiop. The threshold was used because of high prevalence of asymptomatic infections in the populations, as occurs in regions endemic for malaria.

Some explanatory variables are time-dependent and were therefore evaluated for each trimester. These included current age, experience of exposure to other *Plasmodium spp.* (*Plasmodium ovale* and *Plasmodium malariae*) before the current trimester defined by the cumulated number of previous infections, the corresponding year and trimester, time spent in the village during the current trimester. Other variables are individual-dependent including sex, geographic location (e.g. village, house), and genetic profiles (e.g. blood type, hemoglobin type, Glucose-6-phosphate dehydrogenase (G6PD) deficiency status (genotype and Enzyme activity). All variables are summarized in Table 1 and 2.

### Mutation characterization

Sickle cell mutation and alpha-globin 3.7 deletion were typed as described [31]. G6PD mutations and ABO polymorphisms were typed by PCR-RFLP, SNaPshot® (Applied Biosystems, Foster City, USA) or TaqMan SNP genotyping assays (ABI Prism®-7000 Sequence Detection System, Applied Biosystems, Foster City, USA) according to the manufacturer recommendation. Primers, probes and restriction enzymes used are shown in Table 12. PCR conditions will be sent on request. ABO polymorphisms were selected to differentiate the A, B and O alleles [32].

**Table 12:** Primer sequences probes, restriction enzymes and rs numbers used for typing Glucose-6-phosphate dehydrogenase (G6PD) and ABO blood group single nucleotide polymorphisms.

Polymorphism name	rs number	Genotyping method	Forward primer (5′-3′)	Reverse primer (5′-3′)	Probe (5′-3′)	Restriction enzyme
*G6PD*						
G6PD-202	rs1050828	PCR-RFLP	GTGGCTGTTCCGGGATGGCCTTCTG	CTTGAAGAAGGGCTCACTCTGTTTG		*FokI*
G6PD-376	rs1050829	PCR-RFLP	CGTGAATGTTCTTGGTGACG	CCCCAGAGGAGAAGCTCA		*NlaIII*
G6PD-542	rs5030872	TaqMan®	ACCGCATCATCGTGGAGAAG	AGATCTGGTCCTCACGGAACA	probe 1-AGAGCTCTGACCGGCTG	
					probe 2-AGAGCTCTGTCCGGCTG	
G6PD-968	rs76723693	TaqMan®	TGTGGTCCTGGGCCAGTA	GACGACGGCTGCAAAAGT	probe 1-CCAAAGGGTACCTGGACGA	
					probe 2-CAAAGGGTACCCGGACGA	
*ABO*						
ABO-261	rs8176719	PCR-RFLP	GCCTCTCTCCATGTGCAGTA	TCCACAGTCACTCGCCACT		*RsaI*
ABO-297	rs8176720	TaqMan®	TGGCTGGCTCCCATTGTC	CCTGAACTGCTCGTTGAGGAT	probe 1-CGATGTTGAATGTGC	
					probe 2-CGATGTTGAACGTGC	
ABO-467	rs1053878	PCR-RFLP	TGCAGATACGTGGCTTTCCT	CGCTCGCAGAAGTCACTGAT		*EagI*
ABO-526	rs7853989	PCR-RFLP	TGCAGATACGTGGCTTTCCT	CGCTCGCAGAAGTCACTGAT		*BsaHI*
ABO-771	rs8176745	SNaPshot®	CGGGAGGCCTTCACCTAC	CACAAGTACTCGGGGGAGAG	AAAAAACAGTCCCAGGCCTACATCCC	

### HyperCube® data mining algorithm

The HyperCube® technology is accessible as a web based software that requires no specific learning skills, though it requires a significant computing power provided through a SaaS architecture (Institute of Health & Science, Paris, France). A hypercube is a subspace defined by a combination of conditions, each condition being either a range or a modality of a continuous or discrete variable. A hypercube has various characteristics: its dimension, the number of variables involved; the “Lift”, the measure of the over density compared to the whole database, the “Size”, the number of points included in the hypercube.

After defining the dependent variable, HyperCube® program generates a series of rules by exhaustively exploring the space of the random variables, generating optimal subspaces significantly enriched with the occurrence of events, and defining for each interesting subspace, its explicative variables and their corresponding values. A rule is a set of a limited number of continuous and/or categorical variables and their associated values. A search by HyperCube® program is divided in 3 steps:

- *A stochastic exploration of the space of random variables*: Subspaces are exhaustively generated following this procedure: One point is randomly chosen as a germ (a starting point) in the *m*-dimensional space defined by the *m* explanatory variables; after a 2^nd^ point is randomly selected to form a segment. These two points correspond to apical points of a starting subspace having a hypercube design and represent the diagonal of this hypercube. This diagonal (jointly the volume of the hypercube) will be optimally increased. Each subspace is selected depending on two constraints: its size, the number of events included in the subspace, and its purity, the percentage of positive events in the subspace. To define explanatory variables, the corresponding axe for each variable delimiting the subspace is suppressed, and the subsequent subspace tested for satisfying the previous constraints. The variables for which the corresponding axe must be present to satisfy these constraints are the explanatory variables. The subspace is cancelled if it does not satisfy the constraints defined by the user and a new subspace is generated.
- *An optimization of the characteristic of the hypercube*: The volume of each initial hypercube selected at the first step is locally maximized depending on a Z score using genetic algorithms, and always constrained to a minimum purity.
- *Validation of the rule using a non-parametric approach*: The Z score of the optimized hypercube is compared to those generated by a random permutation of the dependant variable.

For exhaustiveness, these three steps are repeated until all points have been used as starting point and all the events have been studied; i.e. all the events in the learning dataset have been included in at least one rule. The user can stop the learning process at any time and know the coverage of his exploration. Due to human limitations in understanding complex rules, the maximal number of explanatory variables inside each rule can be fixed, thereby defining complexity. HyperCube® uses an exhaustive non-parametric and non-Euclidean methodology, it does not use proximity between events but only generates subspaces in which events are present or not.

We have first to define variables to introduce into the learning data set. If necessary, the outcome variable is transformed into a dichotomous variable. In our case, the number of clinical *P. falciparum* attacks by trimester was divided into two groups: “no attack during the trimester”, and “at least one attack during the trimester”. This is done on a local computer using MATRIX program with two main functions: “Simple lift” and “Correlation”. “Simple lift” classifies variables according to their first order effect and has 3 major roles: to verify consistency of the data, to detect circular variables and to detect variables with pivot points that define threshold values for the impact of a variable on the outcome. “Spearman (or Pearson) Correlation” associated with “Simple lift” will help to define which variable to choose amongst the correlated variables. Sometimes, a combined variable from correlated variables is the best choice. The matrix is loaded onto the supercomputer after defining on which part of the database the learning process will be performed. In our case, we chose the learning set of Dielmo cohort. We defined on which group of the dichotomous variable the learning process would be carried out, in our case “at least one attack during the trimester”. First, we constructed a Signal Intensity Graph (SIG), which defines the relationship between the two main parameters of a learning process, “purity” and “size”. This graph shows the value of the “purity” for 5 different “sizes” defined from data of the database and of a randomized database. This graph can be downloaded onto the local computer. After defining the last parameter, “Complexity”, which defines the maximum number of variables per rule, the learning process is run. From the total number of rules, a set of minimized rules is obtained from an iterative process. In the first step, the rule explaining the most number of events is chosen and at each of the following steps the rule explaining the maximal number of events in the remaining event space not included in the first rule is added. The iterative process is stopped when all the events explained by the total number of rules are explained by the set of minimized rules. The total number of rules and/or the minimized rules can be downloaded onto the local computer to perform further analysis.

### Statistical analysis

We used Classification and Regression Trees (CART) methods [5] to split continuous explanatory variables to categories. We performed a Logistic Regression Model to estimate overall RR and OR of combinations of factors [33], [34].

### Identity-by-descent (IBD)

We estimated multipoint IBD using genome wide microsatellite genotypes by MERLIN [35]. We defined “IBD-based mean genetic relatedness” for an individual to the rest of the population, based on IBD probabilities, as the mean of his kinship coefficients with all other individuals = (1/(N−1))×(1/M)×∑_i_ ∑*_m_* [0.5×P1+P2]*_i,m_*, *i* = 1, …, N−1 and *m* = 1, …, M where N is the number of individuals genotyped for the microsatellite markers in the population, M the number of microsatellite markers P1 = probability of sharing 1 allele and P2 = probability of sharing 2 alleles.

### Pedigree-based mean genetic relatedness

The genetic covariance is computed as *r*(A,B) = 2×*coancestry*(A,B) where the *coancestry* between A and B is calculated referring to this following method (Falconer and Mackay 1996) [36]: *coancestry*(A,B) = ∑*_p_*(1/2)*^n(p)^*×(1+I*_Common Ancestor_*) where *p* is the number of paths in the pedigree linking A and B, *n(p)* the number of individuals (including A and B) for each path *p* and I_X_ is the *coancestry* between the two parents of X, which is set to 0 if X is a founder. We defined the mean relatedness coefficient for an individual to the rest of the population, based on the pedigree, as the mean of his kinship coefficients with all other individuals. The variable named “Pedigree-based mean genetic relatedness” was defined by this measure.
