# Correction: An Exhaustive, Non-Euclidean, Non-Parametric Data Mining Tool for Unraveling the Complexity of Biological Systems – Novel Insights into Malaria

**DOI:** 10.1371/annotation/654e34ce-f1cd-4207-b2ac-ebc873b821e9

**Published:** 2011-10-31

**Authors:** Cheikh Loucoubar, Richard Paul, Avner Bar-Hen, Augustin Huret, Adama Tall, Cheikh Sokhna, Jean-François Trape, Alioune Badara Ly, Joseph Faye, Abdoulaye Badiane, Gaoussou Diakhaby, Fatoumata Diène Sarr, Aliou Diop, Anavaj Sakuntabhai, Jean-François Bureau

The authors have the following competing interests to declare: "Author Augustin Huret, writer of the algorithm, sells use of this algorithm through the company Effiscience. This does not alter our adherence to all the PLoS ONE policies on sharing data and materials, as detailed online in the guide for authors." 

